# Efficacy of anti-tuberculosis drugs for the treatment of latent tuberculosis infection: a systematic review and network meta-analysis

**DOI:** 10.1038/s41598-023-43310-8

**Published:** 2023-09-27

**Authors:** Panida Yoopetch, Thunyarat Anothaisintawee, Agampodi Danushi M. Gunasekara, Jiraphun Jittikoon, Wanvisa Udomsinprasert, Montarat Thavorncharoensap, Sitaporn Youngkong, Ammarin Thakkinstian, Usa Chaikledkaew

**Affiliations:** 1 Mahidol University Health Technology Assessment (MUHTA) Graduate Program, Mahidol University, Bangkok, Thailand; 2 Department of Clinical Epidemiology and Biostatistics, Faculty of Medicine Ramathibodi Hospital, Mahidol University, Bangkok, Thailand; 3 Department of Paraclinical Sciences, Faculty of Medicine, General Sir John Kotelawala Defence University, Ratmalana, Sri Lanka; 4 Department of Biochemistry, Faculty of Pharmacy, Mahidol University, Bangkok, Thailand; 5 Social Administrative Pharmacy Division, Department of Pharmacy, Faculty of Pharmacy, Mahidol University, Bangkok, Thailand

**Keywords:** Infectious diseases, Preventive medicine

## Abstract

Despite the availability of three network meta-analyses (NMA) examining the efficacy, treatment completion, and adverse events associated with all latent tuberculosis infection (LTBI) treatments, there is currently no evidence to support the notion that the benefits of these treatments outweigh the potential risks. This NMA aimed to conduct a comprehensive comparison and update of the efficacy, treatment completion rates and adverse events associated with recommended treatment options for LTBI for individuals with confirmed LTBI, as outlined in the 2020 World Health Organization (WHO) Consolidated Guidelines for TB preventive treatment. A comprehensive search of the MEDLINE and Scopus databases was conducted until April 2023. The NMA was applied to estimate the risk difference and corresponding 95% confidence interval (CI) using a combination of direct and indirect evidence. The risk–benefit assessment was employed to evaluate the feasibility of the extra benefits in relation to the extra risks. The primary outcomes of interest in this study were active TB disease, completion rates, and adverse events. The meta-analysis incorporated data from 15 studies, which collectively demonstrated that the administration of a placebo resulted in a significant increase in the risk of developing TB disease by 1.279%, compared to the daily intake of isoniazid for 6 months (6H). Furthermore, treatment completion rates were significantly higher when using isoniazid plus rifapentine weekly for 3 months (3HP) and rifampicin daily for 4 months (4R), as compared to 6H. Considering adverse events, the combination of 3HP, 4R, and isoniazid administered daily for 9 months (referred to as 9H) significantly decreased adverse events by 4.53% in comparison to 6H. The risk–benefit assessment showed that alternative treatment regimens (9H, 4R, 3HR and 3HP) had a lower incidence of adverse events, while demonstrating a higher efficacy in preventing TB, as compared to 6H. This review indicates that there were no significant differences observed among various active treatment options in terms of their efficacy in preventing active TB. Moreover, completion rates were higher in 3HP and 4R, and a reduction in adverse events was observed in 3HP, 4R, and 9H.

## Introduction

Tuberculosis (TB) is a common cause of mortality arising from a solitary infection. In the year 2019, it is estimated that there were approximately 1.2 million deaths worldwide attributed to TB disease^1^. The etiology of TB is caused by *Mycobacterium tuberculosis*. However, a significant proportion of individuals, approximately 90% exhibit no clinical manifestations of the disease, indicating the presence of latent tuberculosis infection (LTBI)^2^. It has been reported that populations with LTBI have an estimated lifetime probability of developing active TB disease of approximately 5% to 10%^3^. As per a meta-analysis conducted in 2019, the global prevalence of LTBI was found to be 24%^4^. The South-East Asia region is widely recognized for having the highest prevalence of LTBI, accounting for 35% of the global burden^5^.

Despite the significant burden of LTBI in Asia, the current approach to treating LTBI is suboptimal, primarily due to low adherence rates and the occurrence of hepatotoxicity as a serious adverse event^6^. According to the 2020 World Health Organization (WHO) Consolidated Guidelines for TB preventive treatment^7^, it is recommended to utilize either a tuberculin skin test (TST) or an interferon-gamma release assay (IGRA) for the purpose of testing for LTBI. Furthermore, the treatment options recommended for the management of LTBI irrespective of the individual's HIV status consist of the following regimens: isoniazid for 6 months daily (6H), isoniazid for nine months daily (9H), isoniazid plus rifampicin for three months daily (3HR), and isoniazid plus rifapentine for three months weekly (3HP). Furthermore, there are alternative treatment regimens available, such as a four-month daily regimen of rifampicin (4R) or a four-week daily regimen of isoniazid plus rifapentine (1HP).

To date, there have been three network meta-analyses (NMA) reporting on the efficacy^8–10^, completion rates^9,10^, and occurrence of adverse events associated with treatment regimens for LTBI^8,10^. Two published NMAs incorporated all individuals receiving LTBI treatment^8,10^. These studies encompassed cases with or without confirmation of LTBI testing. Additionally, another study specifically examined populations with confirmed LTBI, focusing solely on the efficacy and completion rate of LTBI regimens, without investigating adverse events^9^. Moreover, it is worth noting that the treatment regimens mentioned in all the published NMAs encompassed several regimens, including isoniazid for durations ranging from 9 to 72 months and regimens containing pyrazinamide (PZA) for the purpose of indirect comparison. It is important to highlight that these regimens did not currently align with WHO recommended LTBI treatment due to their toxicity. Furthermore, based on the 2020 WHO Consolidated Guidelines^7^, it is imperative to obtain robust evidence to ascertain that the benefits of recommended TB preventive treatment outweigh any potential risks to individuals with LTBI. However, until recently, such evidence has not yet been available. Accordingly, the objective of this NMA was to evaluate whether the extra efficacy (benefit) of LTBI treatment is worth the extra adverse events (risk) using the risk–benefit assessment and to provide an updated analysis of efficacy, treatment completion rates, and adverse events related to LTBI treatments for individuals with confirmed LTBI. These findings would support the current recommendations outlined in the 2020 WHO Consolidated Guidelines.

## Methods

### Study design

This study was conducted in accordance with a PROSPERO-registered protocol, specifically identified as number CRD42020208880, and adhered to the reporting guidelines outlined in the Preferred Reporting Items for Systematic Reviews and Meta-Analyses (PRISMA) statement extension for NMA^11^.

### Search strategy and selection criteria

A systematic search was performed on the MEDLINE database through PubMed and Scopus databases up until April of 2023. The study employed a predefined set of search terms encompassing ‘latent tuberculosis infection’, ‘tuberculosis infection’, ‘isoniazid’, ‘rifampicin’, ‘rifapentine’ and randomized controlled trails (RCTs). There were no restrictions imposed on the language or publication date. The search strategies are described in the appendix Table A1.

The inclusion criteria for this study comprised solely of RCTs involving participants diagnosed with LTBI through either a positive TST or an IGRA. The studies evaluated the efficacy of different treatment regimens for LTBI in terms of their ability to prevent the development of active TB, ensure treatment completion, and minimize adverse events. The treatment regimens under evaluation included 6H, 9H, 3HR, 3HP, 4R, in addition to placebo, a substance or treatment devoid of therapeutic efficacy or absence of treatment. The studies with insufficient data for pooling, despite our efforts to contact the authors, as well as the studies published in languages that were not translatable, were excluded from the analysis.

### Study selection and data extraction

The identified studies were reviewed based on the information presented in the title and abstract of each study by two independent reviewers (PY and DG). In cases where abstracts were insufficient in providing a conclusive determination, full articles were reviewed. Disagreement was resolved by reaching a consensus through discussion with the third reviewer (TA). The study's general information including the first author and publication year, the study characteristics including country, number of participants, follow up time in months, and participant characteristics including age, gender, comorbidity, method of LTBI diagnosis, treatment regimens and treatment duration, outcomes data including active TB disease, completion rates or adverse events were all collected, and the data for pooling were extracted into dichotomous outcomes.

### Quality assessment

The methodological quality of the included studies was assessed using RoB2, the second version of the Cochrane tool for assessing risk of bias in RCTs^12^. This tool consists of five domains: (1) the randomization process, (2) the intended interventions, (3) missing outcome information, (4) outcome measurement, and (5) selection of the reported result. Each item provides a response in the form of “yes” and “probably yes” or “no” and “probably no”. The “no information” response is only provided in cases where there is a lack of sufficient information. Each domain is classified into three categories: low risk of bias, high risk of bias, and some concern. An overall assessment is categorized as follows: (1) High risk of bias if at least one domain is determined to have a high risk, or if multiple domains are determined to have some concern. (2) Some concern if at least one domain is determined to have some concern, but none of other domains is determined to have a high risk of bias. (3) Low risk of bias if the result indicates a low risk of bias for all domains.

### Outcomes of interest

The present study focused on active TB disease as the outcomes of interest. The definition of active TB disease varied across studies, depending on the acceptable standard of TB diagnostic used in each respective study. Some of the criteria used included positive results for *M. tuberculosis* from acid-fast bacilli, sputum smear, clinical suspicion, and radiographic findings.

Adverse events were classified as grade 3, 4, or 5, which included severe outcomes such as death or hepatotoxicity. These events were specifically related to treatment regimens or discontinuation caused by any adverse drug events. The term “grade” pertains to the severity of the adverse events, as defined by the Common Terminology Criteria for Adverse Events (CTCAE)^13^. Grade 3 is considered a serious medical condition, while grade 4 is regarded as extremely dangerous and potentially life-threatening. The completion rates were determined by calculating the percentage of medication doses taken, with the requirement being that participants needed to have taken between 80% and 100% of the prescribed doses for each trial.

### Data synthesis and analysis

A pairwise meta-analysis was performed to evaluate the efficacy of different regimens, focusing on three key outcomes: TB disease, adverse events, and treatment completion. The risk difference (RD), which represents a difference in risk of outcomes between two groups, with 95% confidence interval (CI) of each study was estimated. If there was no heterogeneity between studies, the results were pooled across all included studies using the inverse variance method^14^. If there was heterogeneity among the studies, the pooled RD was calculated using a random-effect model by DerSimonian-laird method^15^.

Heterogeneity was explored by the forest plot and statistical testing. The forest plot is a visual presentation that displays the point estimates of individual studies. If the point estimates are aligned on the same side as the vertical line, which represents the null effect, it is indicative of potential low heterogeneity. If the point estimates of each study are arranged on both sides of a vertical line, it could potentially indicate substantial heterogeneity. The Cochran’s Q test and Higgins’s I^2^ statistic were further used to evaluate the heterogeneity among studies^16^. The Q test yields a p-value that signifies the likelihood of observing such variability due to random chance. A p-value below 0.1 typically indicates a notable degree of heterogeneity^17^. According to commonly used guidelines, Higgins’s I^2^ statistic is interpreted as follows: a range of 0%–25% indicates no heterogeneity, 25%–50% indicates moderate heterogeneity, 50%–75% indicates substantial heterogeneity, and 75%–100% indicates considerable heterogeneity^15^. Based on I^2^ index, when heterogeneity is present (I^2^ > 25%), it is recommended to apply a random effect model^15,16^. If the p-value from Cochrane’s Q test is less than 0.10 or if the Higgins’s I^2^ statistic is greater than 25%, it is inferred that there exists heterogeneity within the data, and a random effect model was applied. Alternatively, if the *p* value obtained from Cochrane’s Q test is greater than 0.10, or the Higgins’s I^2^ statistic is less than 25%, it is assumed that there is no evidence of heterogeneity, and a fixed effect model was applied.

A network meta-analysis was conducted to evaluate and compare the efficacy, completion rates, and occurrence of adverse events among various treatment regimens included in the studies. A two-stage approach was applied. In the initial stage, it is important to estimate the effect size (risk difference) and variance–covariance for dichotomous outcomes in each study. A multivariate random effect meta-analysis with consistency model was applied in the second stage to pool the risk difference data from the various studies. The various effect sizes were contrasted by estimating the relative treatment effects among different active treatment regimens. The probability of being the most effective treatment among all treatment regimens was determined using a rankogram and the surface under the cumulative ranking curve (SUCRA). The Y-axis represents the SUCRA value, a single numerical metric ranging from 0% to 100%, used to evaluate the likelihood of adverse events associated with different treatments in the NMA. The X-axis corresponds to the ranking, ranging from best to worst, based on the SUCRA values. Higher SUCRA values indicate a greater likelihood that a treatment is ranked at the top (best), with values closer to 100% indicating a higher probability. On the contrary, when SUCRA values are lower, approaching 0%, it indicates a higher likelihood that a treatment is positioned in the bottom rank (worst), indicating poorer performance. The consistency assumption was performed by applying the design-by-treatment interaction model to assess design inconsistency. If the *p* value is less than 0.05, it indicates the statistically significant presence of inconsistency. In such cases, a loop-specific approach was employed to identify the treatment arms and studies that contributed to the inconsistency. The source of inconsistency was explored. The comparison-adjusted funnel plot was utilized to address publication bias in NMA.

The risk–benefit assessment was performed to evaluate both the risk (adverse events) and benefits (TB prevention) simultaneously^18^. The risk difference (incremental (Δ)) was estimated through NMA. Afterwards, an incremental risk and benefit ratio (IRBR) was evaluated and simulated by the Monte Carlo simulation with 1000 replications, assuming normal distributions for both incremental risk and incremental benefit. All analyses were performed using STATA software package, version 16.0 (Stata Corp, College Station, Texas, USA).

## Results

**Figure 1:**
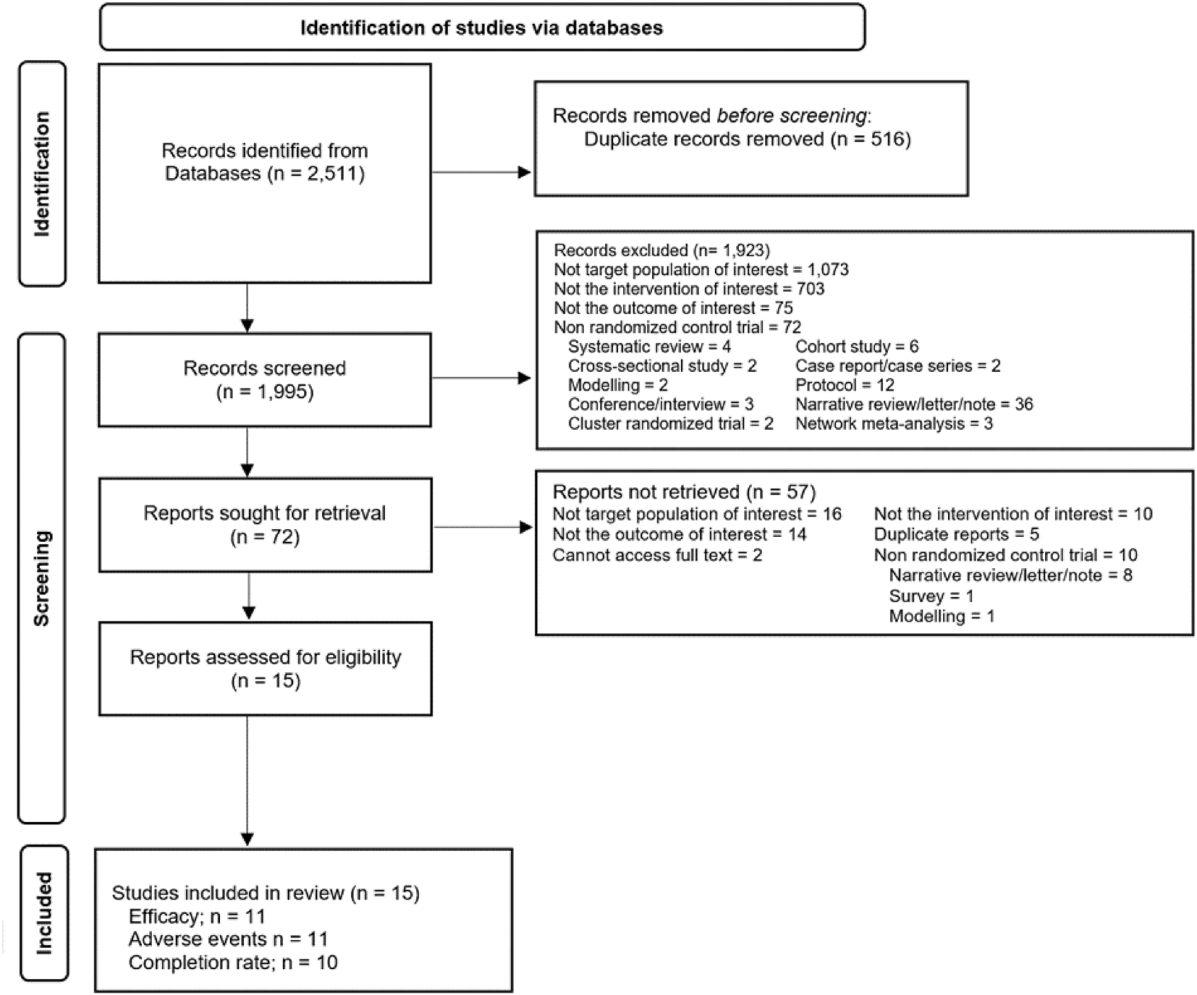
Flow diagram and references of included studies.

A total of 2400 records were identified from two aforementioned databases. Out of these, 72 articles were reviewed in full text as they were deemed potentially relevant. Out of the articles reviewed, 57 were excluded for various reasons. The majority of these exclusions were due to the fact that they did not pertain to the target population of interest (16 articles), did not address the outcomes of interest (14 articles), or did not involve the intervention of interest (10 articles). Additionally, 10 articles were excluded because they were not RCTs, and 7 articles were excluded for other reasons. In summary, a total of 15 studies were included in the present NMA. Of these studies, 11 studies^19–29^, 11 studies^20–22,24–28,30–32^ and 10 studies^19,20,22,24–26,28,29,32,33^ were included in the NMA for efficacy, adverse events and treatment completion, respectively. The PRISMA flow diagram of electronic searching is shown in Fig. 1.

### Characteristics and quality of included studies

The characteristics of included studies are summarized in Table 1. All the included studies were 15 parallel RCTs, that examined six different treatment regimens: 6H, 9H, 4R, 3HR, 3HP, and either a placebo or no treatment. Studies conducted between 1982 and 2018 across various locations. The follow-up time varied between 16 and 60 months on average. Those studies included LTBI participants whose numbers ranged from 96 to 13,955, and whose median age was between 10 and 50 years. The male participation percentage varied between 17% and 100%. Most the included studies (11 studies) diagnosed participants with LTBI using TST, while three studies utilized both TST and IGRA (20) (25) (30). Only one study used IGRA alone^23^. The mode of administration was not reported in most studies (7 studies)^19,23,25,27,29,31,32^. Out of these, five studies reported administration through self-administered therapy (SAT) ^20–22,28,33^, while two studies reported administration through both directly observed therapy (DOT) and SAT^24,26^. Administration by DOT was reported in only one study^30^. Four studies were conducted on populations living with human immunodeficiency virus (HIV)^22,24,27,28^. Additionally, four other studies were conducted in prisoners^30^, immigrants^21^, kidney transplant patients^23^ and children^20^.

**Table 1:** The characteristics of included studies.

Authors	Year	Country	Average follow-up time (months)	% Male	Median age (yr.)	Population features	LTBI diagnosis	Induration (TST)	Administration	Treatment regimen
IUAT	1982	Czechoslovakia, Finland, German Democratic Republic (DDR), Hungary, Poland, Romania, and Yugoslavia	60	53	50	Not report	TST	> 6 mm	Not report	6H Placebo
Chan	2012	Taiwan	Not report	100	Not report	Prison	TST / QFT-GIT	≥ 10 mm	DOT	6H 4R
Diallo	2018	Australia, Benin, Brazil, Canada, Ghana, Guinea and Indonesia	16	49.7	10.2	Children	TST / QFT / T-Spot	not specific	SAT or caretakers	9H 4R
Jiménez-Fuentes	2013	Barcelona, Spain	60	67.8	26.1*	Immigrants	TST	> 5 mm in contacts > 15 mm in other cases	SAT	6H 3HR
Johnson	2001	Uganda	24	31 29 31	29* 29* 30*	HIV	TST	≥ 5 mm	SAT	6H 3HR Placebo
Kim	2015	South Korea	21.7	58	43.9*	Kidney transplant	T-SPOT.TB	NK	Not report	9H Placebo
Martinson	2011	South African	46.8 48	16.7	30.4	HIV	TST	> 5 mm	SAT DOT	6H 3HP
Menzies	2018	Australia, Benin, Brazil, Canada, Ghana, Guinea, Indonesia, Saudi Arabia, and South Korea	28	40.9	38.4*	NK	TST / IGRA	≥ 5 mm, ≥ 10 mm, ≥ 15 mm	Not report	9H 4R
Menzies	2004	Canada	Not report	50 62	34.8* 32.9*	Not report	TST	> 5 mm	SAT SAT	9H 4R
Menzies	2008	Canada, Saudi Arabia and Brazil	Not report	53 52	Not report	Not report	TST	Not specific	not report	9H 4R
Sterling	2011	The United States, Canada, Brazil, and Spain	33	53.5 55.4	35 36	Not report	TST	CDC	SAT DOT	9H 3HP
Sun HY	2018	Taiwan	24	54.2 61.4	32* 31.7*	Not report	TST	Not specific	Not report	9H 3HP
Gordin	1997	The United States	30	68	38*	HIV	TST	< 5 mm	Not report	6H Placebo
Rivero	2007	Spain	24	77 80	31.3 33	HIV	TST	≥ 5 mm	SAT	6H 3HR
Geijo	2007	Spain	60	22 31	44.16 41.38	not report	TST	CDC	NK	6H 3HR

*DOT* Directly observed therapy, *SAT* Self administered therapy, *TST* tuberculin skin test, *QFT-GIT* QuantiFERON®-TB Gold In-Tube test, *6H* isoniazid 6 months daily; *9H* isoniazid 9 months daily; *4R* rifampicin 4 months daily; *3HR* isoniazid plus rifampicin 3 months daily; *3HP* rifapentine plus isoniazid weekly 3 months.

*mean.

The quality of study was evaluated using RoB2^12^. Overall, there were some concerns for nine studies^19,21,23,27–31,33^, high risk for two studies^24,32^, and low risk for four studies^20,22,25,26^. Details on grading of the quality of studies are shown in the appendix Figure A1.

### Treatment outcome

**Figure 2:**
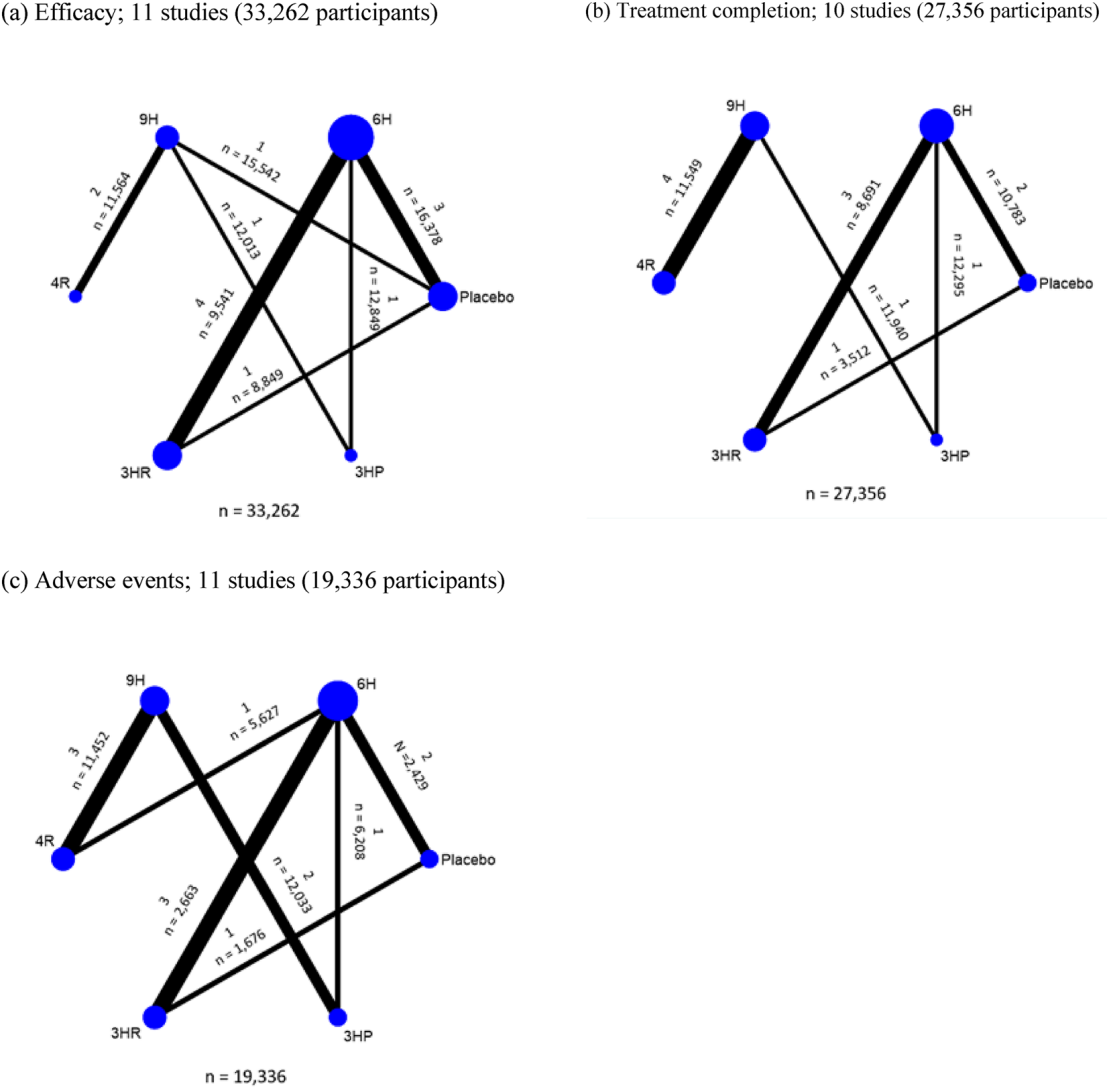
Network comparisons of studies included in the analysis in term of (**a**) Efficacy (**b**) Treatment completion (**c**) Adverse events. The size of the nodes refers to the number of included studies for each comparison, the thickness of the lines refers to the sample size for that comparison. Numbers above and below show number of studies and population. *6H* isoniazid 6 months daily; *9H* isoniazid 9 months daily; *4R* rifampicin 4 months daily; *3HR* isoniazid plus rifampicin 3 months daily; *3HP* rifapentine plus isoniazid weekly 3 months.

The network of eligible comparisons for efficacy, treatment completion, and adverse events outcomes is presented in Fig. 2. The six treatment regimens included in the network map were isoniazid for 6H, 9H, 4R, 3HR, and 3HP.

### Efficacy

#### Pairwise meta-analyses

Regarding the efficacy outcome, it was observed that 6H significantly reduced the risk of TB disease by 0.9% (RD − 0.009, 95% CI − 0.012, − 0.006) compared to placebo. Compared to 3HR, there was a 0.3% increase in the risk of TB disease (RD 0.003, 95% CI − 0.005, 0.012). No statistical heterogeneity was found in these pairwise comparisons, as shown in Table A2 in the appendix.

#### Network meta-analyses

**Figure 3:**
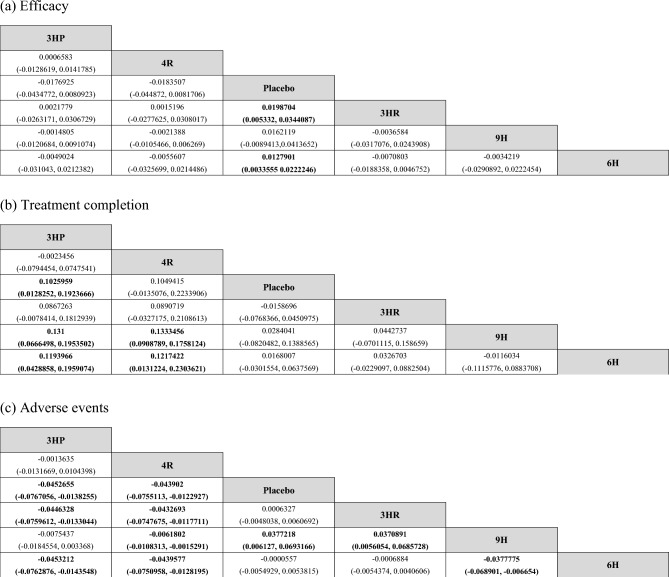
Network estimated risk difference (95% CIs) of treatment options for (**a**) Efficacy, (**b**) Treatment completion and (**c**) Adverse events. Statistically significant differences between treatment options are presented in bold.

The NMA of 11 studies involving 33,262 participants assessed active TB disease of six treatment regimens for LTBI (Fig. 2a). Treatment regimens were compared to the standard treatment, 6H. The analysis showed that the use of placebo resulted in a significant increase in the risk of TB disease by 1.279% (RD 0.0127901, 95% CI 0.0033555, 0.0222246) compared to 6H. There were no significant differences observed for 9H (RD − 0.0034219, 95% CI -0.0290892, 0.0222454), 4R (RD − 0.0055607, 95% CI − 0.0325699, 0.0214486), 3HR (RD − 0.0070803, 95% CI − 0.0188358, 0.0046752) and 3HP (RD − 0.0049024, 95% CI − 0.031043, 0.0212382) when compared with 6H (Fig. 3a).

In terms of TB prevention, the NMA suggested that 3HR and 4R were associated with the highest probability of TB prevention (SUCRA, 0.7), followed by 3HP (SUCRA, 0.6), 9H (SUCRA, 0.5), 6H (SUCRA, 0.4), and placebo (SUCRA, 0.05). The ranking of treatments based on SUCRAs is displayed in the appendix Figure A2a.

A global inconsistency test was performed, and a loop of placebo-6H-3HR presented a significant difference (appendix Table A3). This could be attributed to the fact that the follow-up time for placebo and 3HR was shorter than other studies (22). As time passes, the annual risk of developing active TB from LTBI declines. However, the cumulative risk still increases cumulatively^34^. This implies that studies with a longer follow-up duration should a greater number of cases for treatment comparisons. Subgroup analyses could not be performed due to the limited number of RCTs. The network's comparison-adjusted funnel plots displayed no evidence of asymmetry (the appendix Figure A3a).

### Treatment completion

#### Pairwise meta-analyses

In terms of the treatment completion rate, the use of 6H resulted in a decrease of 2.7% (RD − 0.027, 95% CI − 0.098, 0.045), compared to the use of 3HR. Compared to 4R, 9H resulted in a significant reduction of completion rate by 13.2% (RD -0.132, 95% CI − 0.168, − 0.096). Both exhibited moderate heterogeneity (the appendix Table A2). The observed difference might be attributed to HIV population (6H vs. 3HR). Individuals living with HIV tend to exhibit higher levels of adherence than those who do not have HIV. Regarding the comparison between 9H and 4R, it was observed that there was a difference in adherence among children. The adherence of children with unsupervised regimens tends to be low^35^.

#### Network meta-analyses

The NMA of 10 studies involving 27,356 participants evaluated treatment completion for six treatment regimens for LTBI (Fig. 2b). All regimens were compared to 6H as the standard treatment. The completion rate of 3HP (RD 0.1193966, 95% CI 0.0428858, 0.1959074) and 4R (RD 0.1217422, 95% CI 0.0131224, 0.2303621) was significantly increased by 12% compared with 6H. The study did not find any significant differences in the completion rate when compared the placebo (RD 0.0168007, 95% CI − 0.0301554, 0.0637569), 3HR (RD 0.0326703, 95% CI − 0.0229097, 0.0882504), and 9H (RD -0.0116034, 95% CI − 0.1115776, 0.0883708) with the 6H group (Fig. 3b).

**Figure 4:**
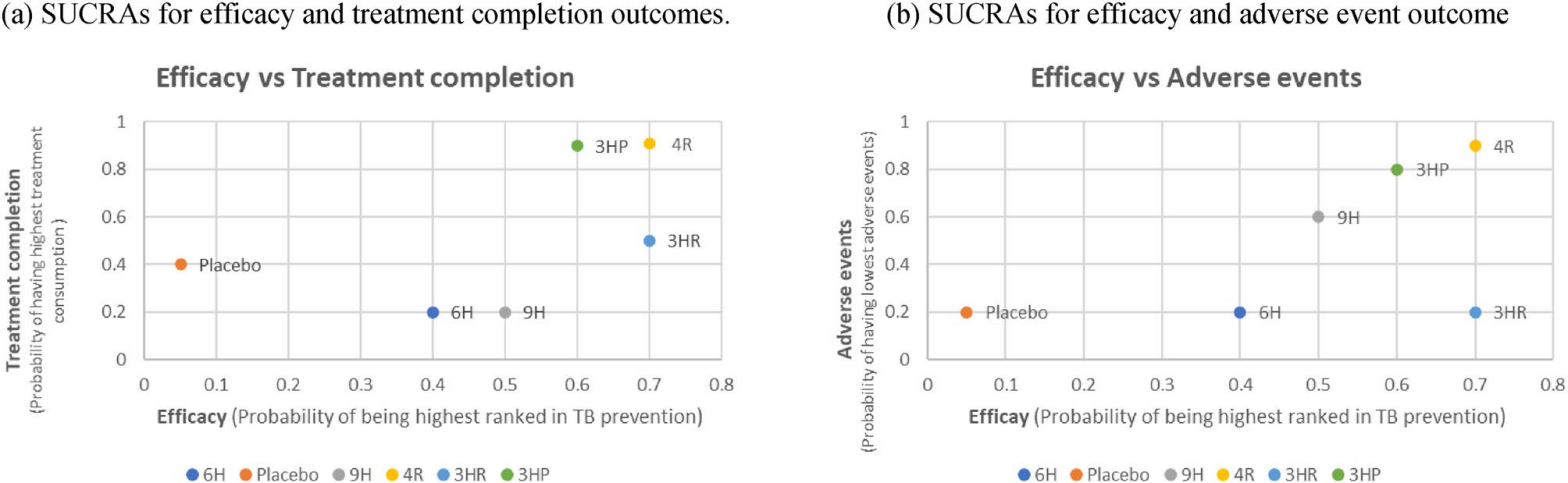
SUCRAs for (**a**) efficacy and treatment completion outcomes. and (**b**) efficacy and adverse event outcomes. SUCRAs (Surface Under the Cumulative Rankings) score from 0 to 1 represent the probability of being best. For efficacy outcome, higher score refers to higher proportion of TB prevention. For treatment completion, higher score corresponds to higher proportion achieving treatment consumption (between 80 and 100% of doses). For adverse event outcome, higher score refers to lower probability of adverse events (Grade 3 to Grade 5).

In terms of treatment completion, findings from the NMA suggest that 3HP and 4R had the highest likelihood of achieving treatment consumption (SUCRA, 0.9), followed by 3HR (SUCRA, 0.5), placebo (SUCRA, 0.4), 6H (SUCRA, 0.2), and 9H (SUCRA, 0.2). The ranking of treatments based on SUCRAs is shown in the appendix Figure A2b. SUCRAs for efficacy and treatment completion outcomes showed that the 4R regimen was linked with the highest levels of efficacy and treatment completion (Fig. 4a).

A test for global inconsistency test was performed, and the results showed no evidence of inconsistency in the treatment network regarding treatment completion (appendix Table A3). Subgroup analyses cannot be performed because of insufficient data. Comparison-adjusted funnel plots for network revealed no evidence of asymmetry (the appendix Figure A3b).

### Adverse events

#### Pairwise meta-analyses

Concerning the adverse events, the use of 6H resulted in a slight increase of 0.3% (RD 0.003, 95% CI − 0.003, 0.010) compared to 3HR. Compared to 4R, 9H resulted in a 1.3% increase in adverse events (RD 0.013, 95% CI 0.007, 0.019). There was no evidence of statistical heterogeneity in these pairwise comparisons (the appendix Table A2).

#### Network meta-analyses

The NMA of 11 studies involving 19,336 participants evaluated adverse events of six treatment regimens for LTBI (Fig. 2c). The adverse events of these treatment regimens were compared to that of 6H, the standard treatment. When compared to 6H, the use of 3HP, 4R, and 9H resulted in a significant decrease in adverse events by 4.53% (RD − 0.0453212, 95% CI − 0.0762876, − 0.0143548), 4.39% (RD − 0.0439577, 95% CI − 0.0750958, − 0.0128195) and 3.77% (RD − 0.0377775, CI − 0.068901, − 0.006654), respectively. There were no significant differences observed between placebo (RD − 0.0000557, 95% CI − 0.0054929, 0.0053815) and 3HR (RD − 0.0006884, 95% CI − 0.0054374, 0.0040606) compared to 6H (Fig. 3c). The efficacy and adverse event outcomes of SUCRAs (Fig. 4b) showed that the 4R regimen was associated with the highest efficacy and the lowest occurrence of adverse events.

A test for global inconsistency was performed, and the result showed no evidence of inconsistency in the treatment network for adverse events (appendix Table A3). However, subgroup analyses cannot be conducted due to a small number of trials. Furthermore, the network's comparison-adjusted funnel plots revealed no evidence of asymmetry (the appendix Figure A3c).

### The risk–benefit assessment

**Figure 5:**
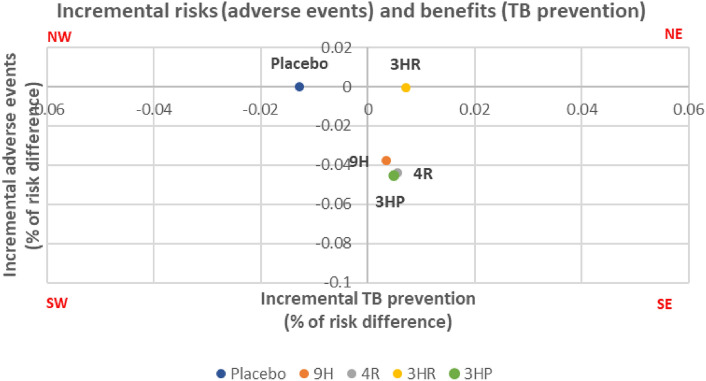
Deterministic results of incremental risks (adverse events) and benefits (TB prevention) ratio on the cost-effectiveness plane.

The risk–benefit assessment was evaluated to simultaneously compare risks (adverse events) and benefits (TB prevention). The NMA estimated the incremental risks and benefits of various treatment regimens (placebo, 9H, 4R, 3HR, and 3HP) compared to 6H. The values for IRBRs were 0.004, − 11.040, − 7.905, − 0.097, and − 9.245 for placebo, 9H, 4R, 3HR and 3HP compared with 6H, respectively (Table 2 and Fig. 5). Alternative treatment regimens (9H, 4R, 3HR and 3HP) were shown to have fewer adverse events and be more effective in preventing TB compared to 6H.

**Table 2:** Deterministic results for ICER of placebo, 9H, 4R, 3HR and 3HP compared with 6H.

Regimen	Incremental risk (adverse events)	Incremental benefit (TB prevention)	IRBR
6H	Reference	Reference	Reference
Placebo	− 0.0001	− 0.0128	0.004
9H	− 0.0378	0.0034	− 11.040
4R	− 0.0440	0.0056	− 7.905
3HR	− 0.0007	0.0071	− 0.097
3HP	− 0.0453	0.0049	− 9.245

**Figure 6:**
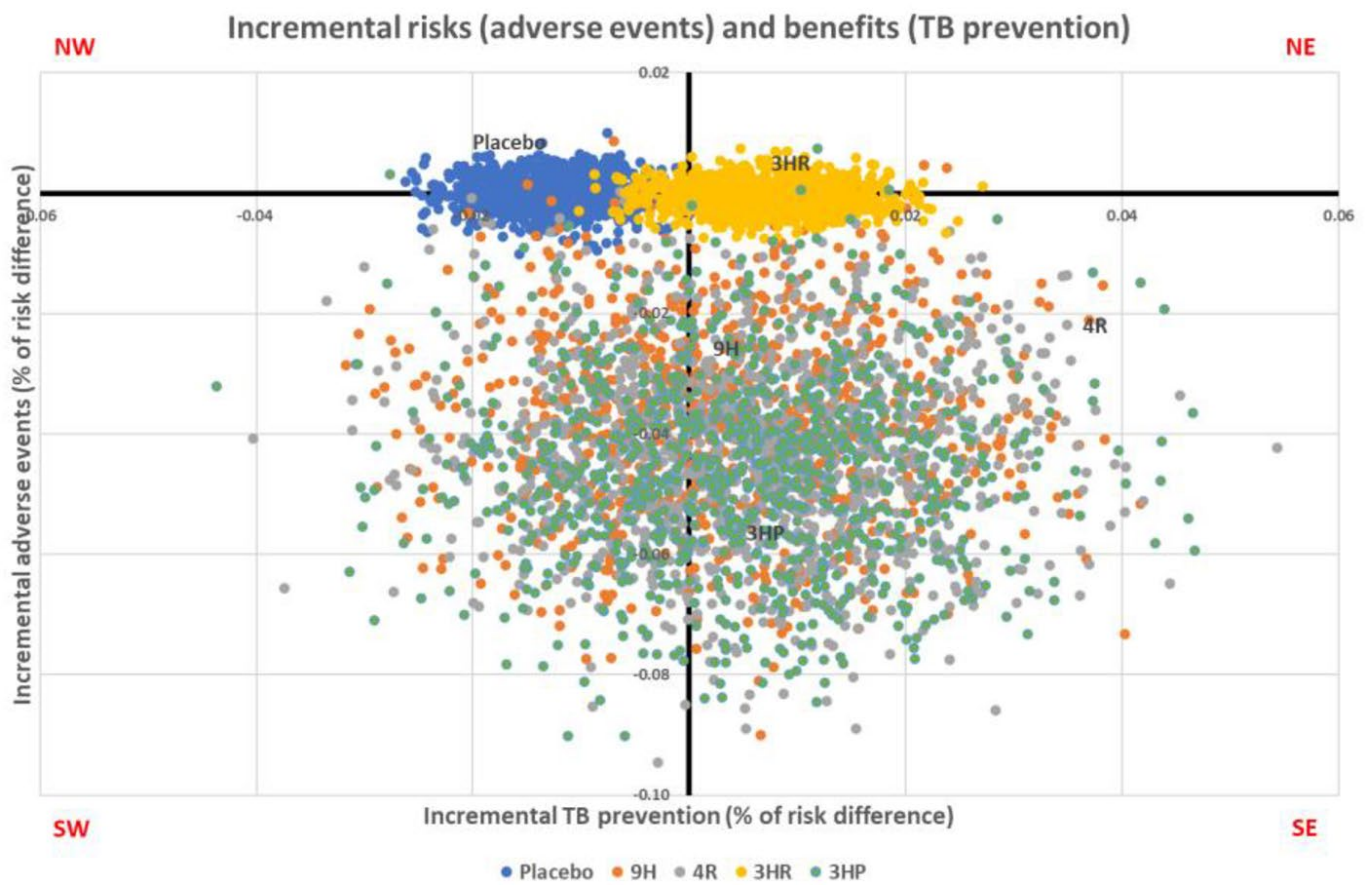
Monte Carlo simulation results of incremental risk and benefit ratios.

The Monte Carlo simulation with 1000 replications was used to simulate incremental risks and benefits, and a cost-effective plane was constructed. The IRBRs for placebo, 9H, 4R, 3HR and 3HP compared to 6H were estimated to be 0.0006, − 9.8737, − 7.2616,  − 0.0807, and − 9.9388, respectively (Table 3 and Fig. 6). The analysis presented that alternative treatment regimens (9H, 4R, 3HR and 3HP) had fewer adverse events and were more effective in preventing TB compared to 6H.

**Table 3:** Monte Carlo simulation for IRBR of placebo, 9H, 4R, 3HR and 3HP compared with 6H.

Regimen	Incremental risk (adverse events)	Incremental benefit (TB prevention)	IRBR
6H	Reference	Reference	Reference
Placebo	− 0.00001	− 0.01296	0.0006
9H	− 0.03795	0.00384	− 9.8737
4R	− 0.04318	0.00595	− 7.2616
3HR	− 0.00057	0.00711	− 0.0807
3HP	− 0.04517	0.00454	− 9.9388

## Discussion

To the best of our knowledge, this was the first NMA to evaluate whether the extra benefits outweigh the extra risks of LTBI regimens using the risk–benefit assessment and to update the efficacy, treatment completion and adverse events of LTBI regimens recommended by the 2020 WHO Consolidated Guidelines for TB preventive treatment among individuals with confirmed LTBI. Based on the Monte Carlo simulation with 1000 replications, the IRBRs for 3HP (− 9.9388), 9H (− 9.8737), 4R (− 7.2616), and 3HR (− 0.0807) suggested that these treatment regimens had higher efficacy and fewer adverse events for those with confirmed LTBI in preventing TB compared to 6H, a standard treatment. In contrast, it was noticed that placebo had less benefit and less risk compared to 6H. This result can be used as the best available evidence to confirm that the benefits of 3HP, 9H, 4R, and 3HR outweigh the risks compared with 6H. This is also in line with the recommendations by the 2020 WHO Consolidated Guidelines for TB preventive treatment^7^ and can assist in making a well-informed decision on trade-offs between the advantages of TB prevention and unfavorable adverse events that must be taken into account prior to starting the treatment.

Unlike two previously published NMA which included all participants receiving available LTBI regimens either with or without LTBI testing, this NMA included only those with confirmed LTBI to ensure that these participants should be given TB preventive therapy. Although one published NMA in 2017 included 31 studies consisting of individuals with confirmed LTBI, they analyzed only efficacy and treatment completion of all LTBI treatment including PZA and isoniazid for longer than 9 months which are not currently recommended by the 2020 WHO Consolidated Guidelines^7^, the National Tuberculosis Controllers Association (NTCA) and the 2020 Centers for Disease Control and Prevention (CDC)^36^ due to drug toxicity. Therefore, of all 31 studies in a previously published NMA^9^, 21 studies were not included due to our exclusion criteria and we added 5 new studies^20,25,27,28,31^, resulting in a total of 15 studies investigating efficacy, treatment completion and adverse events of LTBI treatment recommended by the guidelines among individuals with confirmed LTBI.

In terms of TB prevention, compared with 6H, no significant differences in the efficacy of LTBI regimens were observed for 9H (RD − 0.0034219, 95% CI − 0.0290892, 0.0222454), 4R (RD − 0.0055607, 95% CI − 0.0325699, 0.0214486), 3HR (RD − 0.0070803, 95% CI − 0.0188358, 0.0046752) and 3HP (RD − 0.0049024, 95% CI − 0.031043, 0.0212382). Similar results were also found in the study of Pease et al. indicating that 9H, 4R, 3HR, 3HP, and 6H did not have significant differences in efficacy^9^. Nonetheless, two previously published NMA revealed that all LTBI regimens were more efficacious compared with placebo^8,10^. It should be highlighted that clinicians should consider not only the efficacy of LTBI regimens, but also the characteristics of individuals such as age, medication adherence, risk of adverse events, co-morbidity, and individual preferences should be taken into account^7^.

Moreover, it was observed that shorter regimens lasting for 3–4 months exhibited a higher rate of completion. We found that the completion rates of 3HP (RD 0.1193966, 95% CI 0.0428858, 0.1959074) and 4R (RD 0.1217422, 95% CI 0.0131224, 0.2303621) were significantly increased by 12% compared with 6H. This is consistent with the results from two previously published NMA, showing that the regimens of 3–4 months had a higher chance of regimen completion^9,10^. In addition, there was no significant difference in completion rates between the 3HR and 6H groups. Although a poor adherence could lead to reduced benefit of TB prevention, there is no evidence to suggest that 3HR treatment is associated with reduced benefits. Several factors may also affect adherence, including the method of administration like SAT, DOT, drug tolerance, dose frequency (daily or weekly), and daily pill burden. The explanation for this distinction is likely owing to the fact that the 3HR regimen requires a higher daily pill intake and more frequent dosing compared to the 3 to 4-month regimens of 3HP and 4R.

It should be emphasized that the risk of adverse events should be carefully monitored together with the potential benefit of LTBI regimens^7^. This study was the first NMA that compared the occurrence of adverse events among participants with confirmed LTBI who received LTBI regimens. It was discovered that the use of 3HP, 4R, and 9H resulted in a statistically significant decrease in adverse events by 4.53% (RD − 0.0453212, 95% CI − 0.0762876, − 0.0143548), 4.39% (RD − 0.0439577, 95% CI − 0.0750958, − 0.0128195) and 3.77% (RD − 0.0377775, CI − 0.068901, − 0.006654) compared to 6H, respectively. However, no significant differences in adverse events observed between placebo (RD − 0.0000557, 95% CI − 0.0054929, 0.0053815) and 3HR (RD − 0.0006884, 95% CI − 0.0054374, 0.0040606) compared to 6H. Our findings were different from the study of Assefa et al. published in 2023 which revealed that adverse events were occurred in 3HR treatment group and the risk of hepatotoxicity was significantly higher in 9H followed by 6H compared to placebo, while the other previous NMA did not have data available on the hepatotoxicity of isoniazid and rifampicin regimens^8^.

Our results indicated that the administration of 3HP, 4R and 9H led to a significant reduction in adverse events as compared to 6H. Despite a longer treatment duration, 9H significantly reduced the occurrence of adverse events. It can be explained that LTBI patients receiving 9H tended to have a lower risk of adverse events than those receiving 6H. It is worth noting that the latter group consisted most patients who were also HIV-positive. Given that HIV infection is one of the risk factors attributable to idiosyncratic drug-induced liver injury (DILI), people living with HIV receiving multiple treatments for both HIV and opportunistic infection treatments might suffer from toxicity related to the interaction of drug–drug and drug–disease^37^.

However, our reported risk differences seem relatively small in magnitude even though these differences were statistically significant. It is worth noting that the small extent of these risk differences should be interpreted with the consideration of their clinical significance which will determine whether the results of the RCTs are likely to reflect the impact on real clinical practice^38^. In addition to these risk differences, number needed to treat (NNT) to prevent one event calculated as the inverse of the absolute risk difference, has been applied to assess clinical significance^39^. It is also highlighted that the established threshold exists for NNT which are generally considered as clinically significant when NNT is less than 50 for treatment interventions^39^. Consequently, our results indicated that 6H significantly decreased the risk of TB disease by 0.9% (NNT = 11) compared to placebo. Compared to 6H, the completion rates of 3HP and 4R were significantly increased by 12% (NNT = 8), while the adverse events of 3HP, 4R, and 9H significantly decreased by 4.53% (NNT = 22), 4.39% (NNT = 23) and 3.77% (NNT = 27), respectively. Given that the reported NNT values meet such threshold, our findings can shed light on the practical implications of these results in clinical practice.

It was noteworthy to address the limitations in our review. Firstly, it is important to note that the standard treatment regimens are different, and standard practice is changeable over time. According to published reports on the toxicity profile of PZA, it is recommended that PZA should be restrictedly used^40^. For that reason, we excluded studies containing regimens with PZA to confirm that included studies were comparable interventions. Nevertheless, this could result in less included studies which could lead to potential bias. Therefore, additional RCTs are required to validate this finding and enhance statistical power in future studies. Secondly, we found that the studies included in our review had varying lengths of follow-up time and measured efficacy outcomes related to TB prevention, which may have long-term benefits. However, due to these differences in follow-up time, our review was unable to account for them. Therefore, future studies require more evidences for adverse event outcomes, considering different follow-up time for efficacy outcome and incorporating other factors such as HIV positive patients receiving antiretroviral therapy. Additionally, the recent evidence for a shorter duration of treatment, a 1-month regimen of rifapentine plus isoniazid should be included.

In conclusion, our risk–benefit assessment suggests that 9H, 4R, 3HR and 3HP had higher efficacy and fewer adverse events than 6H, the standard treatment. This information could be applied to support the current clinical practice for TB preventive treatment. Moreover, this comprehensive review summarized the effect of LTBI treatment regimens on efficacy, completion rate, and adverse events. According to the NMA, there was no significant difference in the prevention of active TB among the various active treatment options. Additionally, it was found that treatment regimens 3HP and 4R had a higher completion rate. Furthermore, regimens 3HP, 4R, and 9H showed a decrease in the occurrence of adverse events. Considering on efficacy, completion rate, adverse events, and risk–benefit assessment results, the treatment regimens 3HP and 4R would be highly recommended for individuals with confirmed LTBI. This information could be used to support the recommendation from the 2020 WHO Consolidated Guidelines and assist clinician and public health program management for TB preventive treatment.

### Supplementary Information

Supplementary Information 1.

Supplementary Information 2.

Supplementary Information 3.

## Data Availability

All data generated or analyzed during this study are included in this published article.
