# Supplementary material for: Efficacy of anti-tuberculosis drugs for the treatment of latent tuberculosis infection: a systematic review and network meta-analysis
Source: Sci Rep. 2023 Sep 27;13:16240. doi: 10.1038/s41598-023-43310-8 (PMC10533889; doi:10.1038/s41598-023-43310-8)
Supplement: Supplementary file 3 — Supplementary Information 3. [file 41598_2023_43310_MOESM3_ESM.docx]

**Appendix**

**Appendix Table A1: Search strategies**

**Database: Pubmed**

| **Pubmed (April, 2023)** | **No** | **Query** |
| --- | --- | --- |
| (P) Disease/condition | 1 | Tuberculo* |
|  | 2 | tb |
|  | 3 | laten* tubercul* |
|  | 4 | ltbi |
|  | 5 | or/1-4 |
| (I) Intervention /  (C) Comparator | 6 | Isoniazid prophylaxis |
|  | 7 | Isonia* prophylax* |
|  | 8 | isoniazid preventive therapy |
|  | 9 | ipt |
|  | 10 | chemoprevent* |
|  | 11 | chemoprophyla* |
|  | 12 | Isonia* |
|  | 13 | isonicotinic acid hydrazide |
|  | 14 | INH |
|  | 15 | 9H |
|  | 16 | rifapentine |
|  | 17 | rpt |
|  | 18 | 3HP |
|  | 19 | rifam* |
|  | 20 | rif |
|  | 21 | rmp |
|  | 22 | or/6-21 |
|  | 27 | #5 AND #22 |
|  | 28 | Filters; Humans |
|  | 29 | Filters; RCT |

**Database: Scopus**

| **Scopus (April, 2023)** | **No** | **Query** |
| --- | --- | --- |
| (P) Disease/condition | 1 | TITLE-ABS-KEY ( tuberculo* ) |
|  | 2 | TITLE-ABS-KEY ( tb ) |
|  | 3 | TITLE-ABS-KEY ( laten*  AND tubercul* ) |
|  | 4 | TITLE-ABS-KEY ( ltbi ) |
|  | 5 | or/1-4 |
|  | 6 | TITLE-ABS-KEY ( "Isonia* prophylax*" ) |
|  | 7 | TITLE-ABS-KEY ( "isoniazid preventive therapy" ) |
|  | 8 | TITLE-ABS-KEY ( ipt ) |
|  | 9 | TITLE-ABS-KEY ( chemoprevent* ) |
|  | 10 | TITLE-ABS-KEY ( chemoprophyla* ) |
|  | 11 | TITLE-ABS-KEY ( isonia* ) |
|  | 12 | TITLE-ABS-KEY ( "isonicotinic acid hydrazide" ) |
| (I) Intervention/ (C) Comparator | 13 | TITLE-ABS-KEY ( inh ) |
|  | 14 | TITLE-ABS-KEY ( 9h ) |
|  | 15 | TITLE-ABS-KEY ( rifapentine ) |
|  | 16 | TITLE-ABS-KEY ( rpt ) |
|  | 17 | TITLE-ABS-KEY ( 3hp ) |
|  | 18 | TITLE-ABS-KEY ( rifam* ) |
|  | 19 | TITLE-ABS-KEY ( rif ) |
|  | 20 | TITLE-ABS-KEY ( rmp ) |
|  | 21 | or/6-22 |
| Study designs | 22 | TITLE-ABS-KEY ( "randomized controlled trial" ) |
|  | 23 | #5 AND #23 AND #24 |

**Appendix Figure A1:** Summarized risk of bias of included studies

**Risk of bias summary**


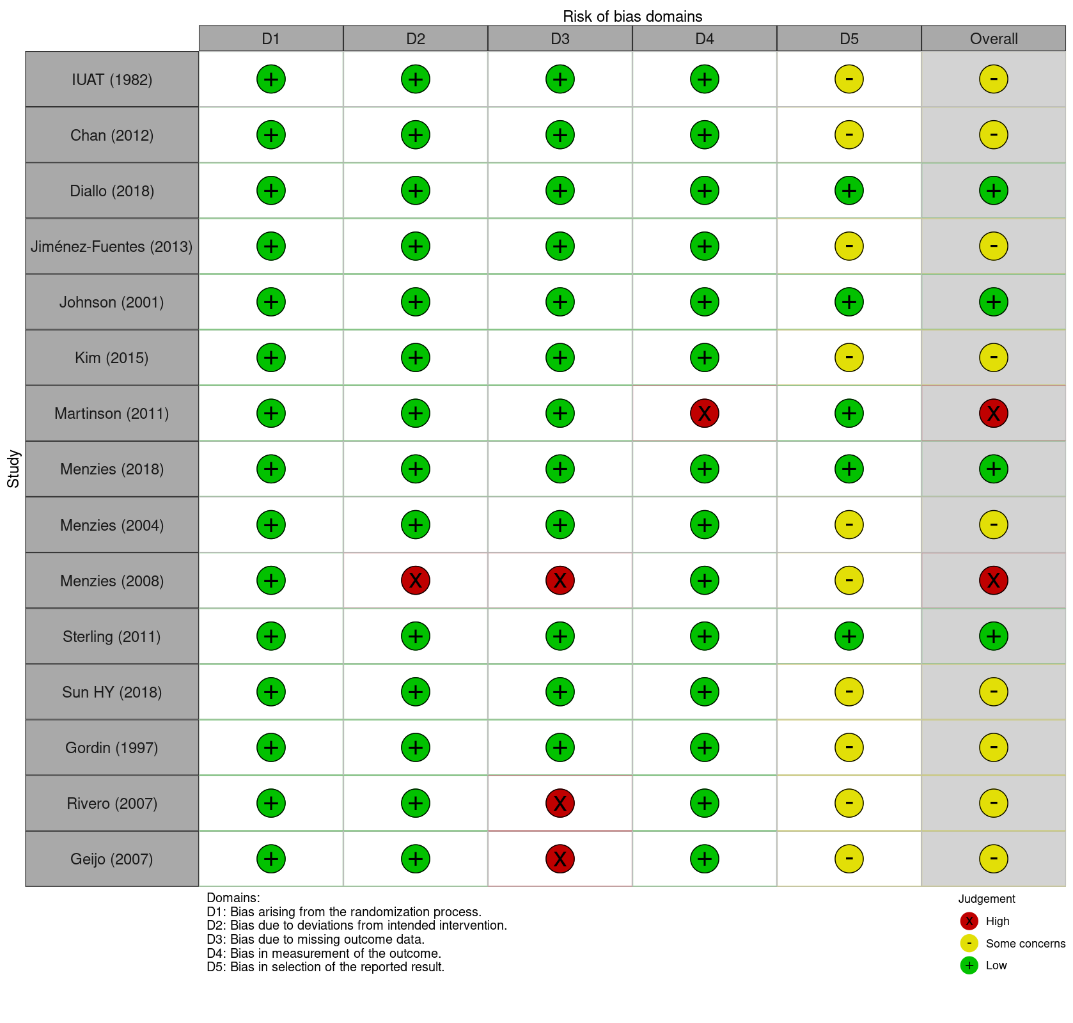


**Risk of bias** **graph**


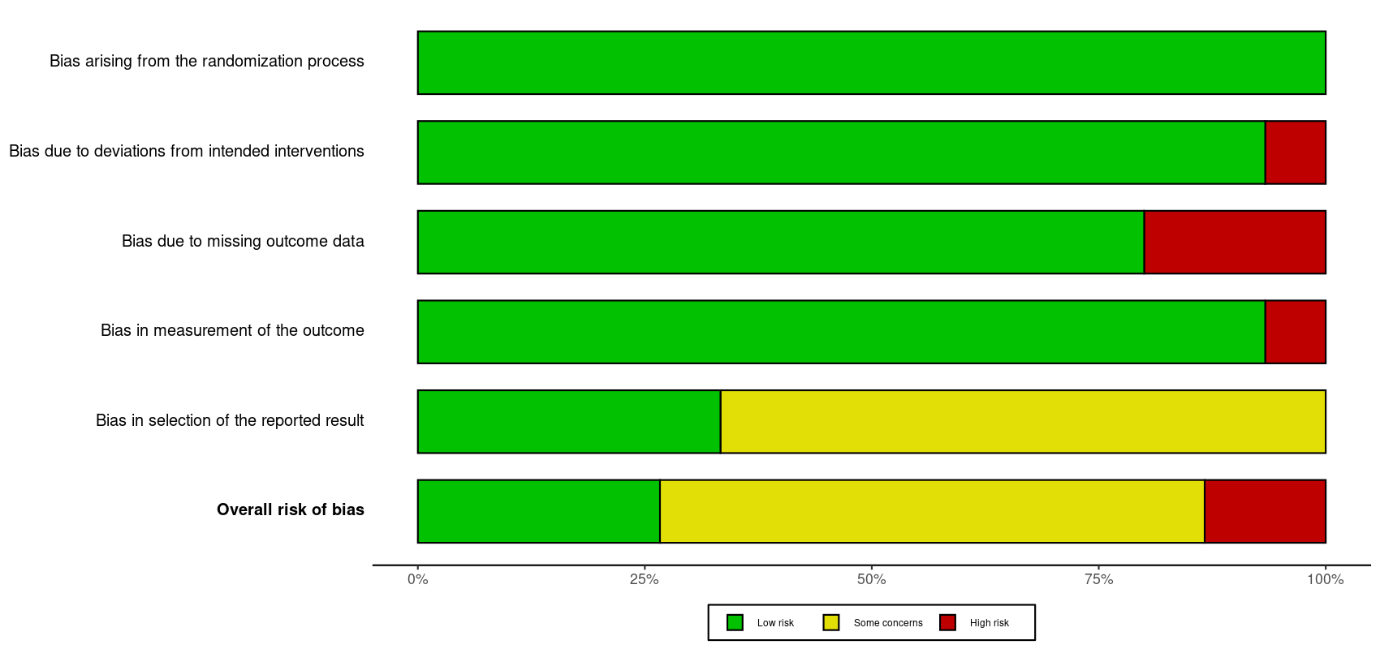


**Appendix Table A2:** Pairwise meta-analysis risk difference (and 95%CI) for dichotomous outcomes.

| **Comparisons** |  | **No. studies** | **Pairwise meta-analysis**  **risk Difference (95%CI)** | **Heterogeneity**  **(I^2^)** |
| --- | --- | --- | --- | --- |
| Efficacy |  |  |  |  |
| 6H | Placebo | 3 | -0.009 (-0.012, -0.006) | 0.00% |
|  | 3HR | 4 | 0.003 (-0.005, 0.012) | 22.09% |
|  | 3HP | 1 | -0.006 (-0.045, 0.033) | NA |
| 9H | Placebo | 1 | -0.023 (-0.048, 0.003) | NA |
|  | 4R | 2 | 0.001 (-0.002, 0.005) | 36.48% |
|  | 3HP | 1 | 0.002 (-0.000, 0.005) | NA |
| 3HR | Placebo | 1 | -0.051 (-0.082, -0.020) | NA |
| Completion |  |  |  |  |
| 6H | Placebo | 2 | -0.015 (-0.068, 0.039) | 86.03% |
|  | 3HR | 3 | -0.027 (-0.098, 0.045) | 41.05% |
|  | 3HP | 1 | -0.119 (-0.165, -0.074) | NA |
| 9H | 4R | 3 | -0.132 (-0.168, -0.096) | 44.49% |
|  | 3HP | 1 | -0.131 (-0.150, -0.112) | NA |
| 3HR | Placebo | 1 | 0.027 (-0.007, 0.062) | NA |
| ADR |  |  |  |  |
| 6H | Placebo | 2 | -0.008 (-0.047, 0.031) | 0.00% |
|  | 4R | 1 | 0.061 (0.016, 0.106) | NA |
|  | 3HR | 3 | 0.003 (-0.003, 0.010) | 0.00% |
|  |  | 2 | 0.003 (-0.003, 0.010) | 0.00% |
|  | RPT | 1 | 0.031 (-0.011, 0.072) | NA |
| 9H | 4R | 3 | 0.013 (0.007,0.019) | 0.00% |
|  | 3HP | 2 | 0.009 (-0.002, 0.019) | 0.00% |

**Appendix Figure A2.** SUCRA ranking curve

(a) Efficacy

40%

70%

60%

70%

50%

5%

(b) Treatment completion

90%

50%

90%

20%

40%

20%

(c) Adverse events

90%

20%

90%

60%%

20%%

20%%

**Appendix Table A3**. Assessment of inconsistency in network meta-analysis

(a) Design-by-treatment inconsistency

| **Network outcome** | **Chi-square** | **P-value for test of global inconsistency** |
| --- | --- | --- |
| Efficacy | 7.64 | 0.0541 |
| Treatment completion | 1.24 | 0.5378 |
| Adverse events | 0.97 | 0.8076 |

(b) A loop-specific

| **Loop of evidence** | **IF (95% CI)** | **P value** |
| --- | --- | --- |
| **Efficacy** |  |  |
| Placebo-6H-3HR | 0.041 (0.01,0.07) | 0.012 |
| Placebo-6H-9H-3HP | 0.021 (0.00,0.07) | 0.390 |
| **Treatment completion** |  |  |
| Placebo-6H-3HR | 0.014 (0.00,0.19) | 0.883 |
| **Adverse events** |  |  |
| 6H-9H-4R-3HP | 0.030 (0.00,0.09) | 0.357 |
| Placebo-6H-3HR | 0.003 (0.00,0.01) | 0.546 |

**Appendix Figure A3.** Comparison-adjusted funnel plot for network.

(a) Efficacy

(b) Treatment completion

(c) Adverse events
